# Zebrafish as a Model to Investigate Dynamin 2-Related Diseases

**DOI:** 10.1038/srep20466

**Published:** 2016-02-04

**Authors:** Cinzia Bragato, Germano Gaudenzi, Flavia Blasevich, Giulio Pavesi, Lorenzo Maggi, Michele Giunta, Franco Cotelli, Marina Mora

**Affiliations:** 1 Neuromuscular Diseases and Neuroimmunology Unit, IRCCS Neurological Institute C. Besta, Milano, Italy; 2 Department of Biosciences, University of Milan, Via Celoria, 26, 20133, Milan, Italy

## Abstract

Mutations in the dynamin-2 gene (*DNM2*) cause autosomal dominant centronuclear myopathy (CNM) and dominant intermediate Charcot-Marie-Tooth (CMT) neuropathy type B (CMTDIB). As the relation between these *DNM2*-related diseases is poorly understood, we used zebrafish to investigate the effects of two different *DNM2* mutations. First we identified a new alternatively spliced zebrafish *dynamin-2a* mRNA (*dnm2a*-v2) with greater similarity to human *DNM2* than the deposited sequence. Then we knocked-down the zebrafish *dnm2a*, producing defects in muscle morphology. Finally, we expressed two mutated *DNM2* mRNA by injecting zebrafish embryos with human mRNAs carrying the R522H mutation, causing CNM, or the G537C mutation, causing CMT. Defects arose especially in secondary motor neuron formation, with incorrect branching in embryos injected with CNM-mutated mRNA, and total absence of branching in those injected with CMT-mutated mRNA. Muscle morphology in embryos injected with CMT-mutated mRNA appeared less regularly organized than in those injected with CNM-mutated mRNA. Our results showing, a continuum between CNM and CMTDIB phenotypes in zebrafish, similarly to the human conditions, confirm this animal model to be a powerful tool to investigate mutations of *DNM2 in vivo*.

Dynamin-2 (*DNM2*) related diseases are a heterogeneous group of conditions that affect muscular and nervous systems. Mutations in *DNM2* cause centronuclear myopathy (CNM), a rare hereditary disease characterized by centrally located nuclei in muscle fibres. Single (autosomal dominant) mutations in *DNM2* occur in around 50% of patients with CNM. Other mutations in *DNM2* cause the dominant intermediate axonal form of Charcot-Marie-Tooth neuropathy type B (hereafter CMT), a motor and sensory neuropathy that primarily affects peripheral nerves^1^,^2^,^3^,^4^.

DNM2 belongs to a large family of cytosolic GTPases that act mechanically and enzymatically to mediate membrane fission. Dynamins and dynamin-like proteins are involved in the budding off of transport vesicles, in organelle division, in cytokinesis and in pathogen resistance^5^. Dynamin-2 is expressed ubiquitously in mammals, dynamin-1 occurs mainly in neurons and dynamin-3 occurs mainly in brain and testes.

Dynamins 1, 2 and 3 have a 5-domain structure: an N-terminal GTPase domain, a middle domain (MD), a pleckstrin-homology (PH) domain that binds phosphoinositides, a GTPase effector domain (GED) that (together with the MD) is involved in oligomerization and regulation of GTPase activity, and a C-terminal proline-rich domain (PRD) that interacts with SH3 domains^6^.

Mutations in the PH domain of dynamin-2, which specifically binds phosphatidylinositol-4, 5-bisphosphate to mediate localization on the membrane, are responsible for severe forms of both CNM and CMT^7^,^8^.

Several pathogenic mechanisms related to DNM2 function have been suggested^9^; however, despite the knowledge gained so far, the pathogenic mechanisms that cause CNM versus CMT are still unknown.

We used zebrafish to investigate and compare the effects of two different *DNM2* mutations, one related to CNM and one to CMT.

Two co-orthologs of human *DNM2* are present in zebrafish, currently named *dnm2a* and *dnm2b* (respectively *dnm2* and *dnm2-like* for Gibbs *et al.* 2013): *dnm2a* is positioned on chromosome 3, while *dnm2b* is on chromosome 1. They are both expressed throughout early development and in all adult tissues, and are required for normal zebrafish development^10^.

After confirming, by whole mount *in situ* hybridization (WISH), that *dnm2a* was the gene linked to the muscular system in zebrafish, but not *dnm2b* (manuscript in preparation), we noticed that the *dnm2a* deposited sequence was incomplete, and therefore performed in silico analysis. This led us to identify a previously unknown alternatively spliced mRNA sequence of the zebrafish *dnm2a*, that we called *dnm2a-*v2, with greater similarity to human *DNM2* than the deposited sequence, that we called *dnm2a-*v1.

We next investigated the effects of *dnm2a* knockdown using two different morpholinos and the effects of rescuing the resulting phenotypes by injecting either the *dnm2a*-v1 or the *dnm2a*-v2 transcript, through assessment of motor behaviour and animal morphology in developing zebrafish embryos.

Finally, and most importantly, we assessed the effects of over-expressing two mutations in the PH domain of human dynamin-2, the R522H mutation responsible for CNM, and the G537C mutation responsible for CMT. In these CNM and CMT models we evaluated motor behaviour, muscle morphology and motor neuron morphology.

## Results

### Two *dnm*2a isoforms in zebrafish

Using publicly available sequences (NCBI, ENSEMBL, ZFIN) we identified a *dnm2* transcript that differed from the one previously reported^10^. The originally identified full-length transcript (RefSeq NM_001030128) lacks a 3′ portion corresponding to the last two exons of human *DNM2*. This was surprising given that homologous genes generally have a highly conserved structure (number and size of exons) across vertebrate classes. Furthermore, among the ESTs mapping to this locus, several were further spliced and extended the 3′ end of the transcript; however only the CV482233 sequence was comparable with the 3′ sequence of the human gene found in RefSeq.

**Figure 1:**
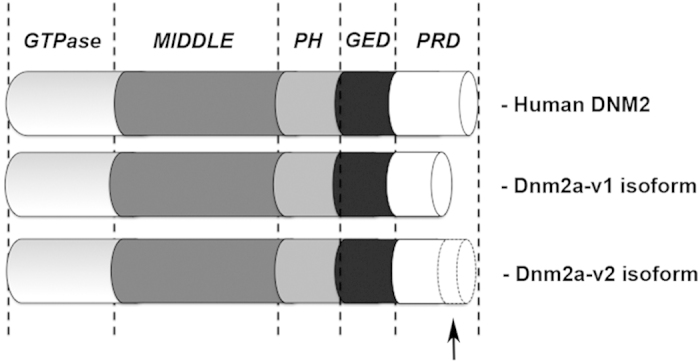
Full-length *dnm2a* mRNA. Protein structure of zebrafish Dnm2a-v1 and Dnm2a-v2 compared to human DNM2. The PRD domain of Dnm2a-v2 is 102 aa longer than Dnm2a-v1 (black arrow), showing greater similarity with human DNM2.

We therefore assembled a sequence from the available ESTs to obtain a transcript, deposited in GenBank (KC968470), that had greater similarity to the human gene sequence than the initially deposited sequence (NM_004945.3). That this sequence was present *in vivo* was shown by 3′RACE determinations on zebrafish RNA extracted from different stages of development (oocites, 2 cells, 32 cells, 50% epiboly, sphere, 128 cells, 12 somites, 24 hpf and 4dpf). Since the new transcript was longer than the previously available RefSeq, we called it *dnm2a-*v2, renaming the original RefSeq transcript *dnm2a*-v1 (Fig. 1). The corresponding proteins were closely similar to human dynamin-2: *dnm2a*-v1 (755 amino acids) was 88% identical, and *dnm2a*-v2 (856 amino acids) was 87% identical. RT-PCR of mRNAs at different developmental stages (Supplementary Fig. 1) showed that, while the *dnm2a*-v1 transcript was present from the earliest stages, *dnm2a*-v2 was not present before the epiboly stage^11^,^12^.

### Zebrafish *dnm2a* expression is related to somite formation

We investigated the spatial localization of *dnm2a* using whole mount *in situ* hybridization (WISH), using a specific probe recognizing a 456 bp region shared by the original *dnm2* (*dnm2a*-v1) and the *dnm2a*-v2 transcript.

**Figure 2:**
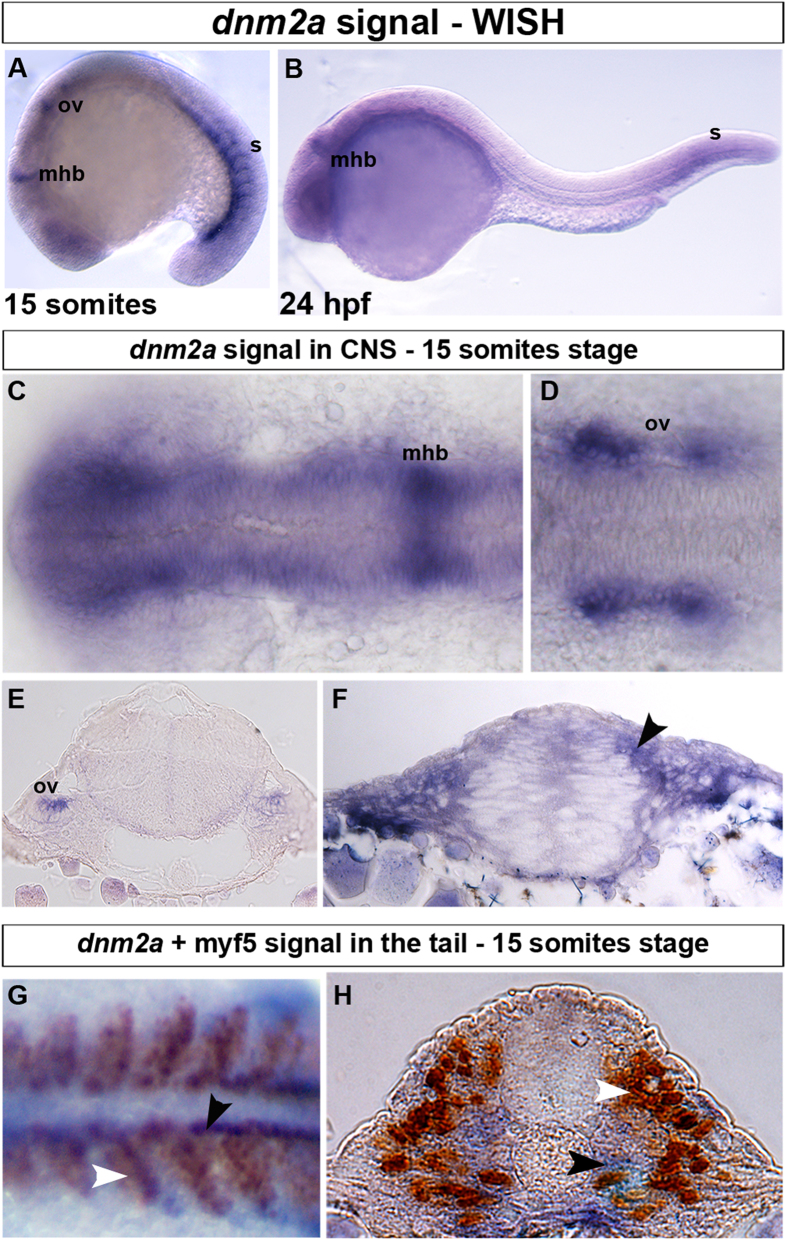
WISH and double-staining of *dnm2a* and myf5. WISH shows that *dnm2a* transcripts are present in the CNS and tail of zebrafish embryos from early somitogenesis (11 hpf approximately) to 30 somites stage (24 hpf). (**A**) At the 15-somite stage *dnm2a* is present in the tail (somites, s), at the midbrain/hindbrain boundary (mhb) and, bilaterally, at the two otic vesicles (ov). (**B**) At 24 hpf, *dnm2a* is present in newly formed somites and appears to be declining in intensity at the midbrain/hindbrain boundary. (**C**) WISH shows *dnm2a* signal in CNS, in embryos at 15 somites stage, more pronounced in the midbrain/hindbrain boundary and in the two bilateral otic vesicles (**D,E**). (**F**) The expression of *dnm2a* is diffuse in the neural tube, and more intense in the periventricular and in the dorso-lateral portion (black arrowhead). (**G**) Double-staining reveals *dnm2a* and myf5 at the 15 somite stage: *dnm2a* appears (in dorsal view of flat mounted embryo *dnm2a* in somites) posteriorly and close to the notochord (black arrowhead) overlapping to some extent with myf5 (white arrowhead); in cross section (**H**), *dnm2a* is present in the medio-ventral part of the somite and in several adaxial cells (black arrowhead), while myf5 is expressed in medial cells (white arrowhead).

We detected *dnm2a* from early somitogenesis, 11 hours post fertilization (hpf) approximately, to 30 somites stage (24 hpf), in specific areas of the CNS and tail (Fig. 2). In the CNS, *dnm2a* was expressed at the midbrain-hindbrain boundary (Fig. 2A,C) and in the bilateral otic vesicles (Fig. 2A,D,E), with a diffuse signal in the neural tube, and a strong positivity in the periventricular and in the dorso-lateral portion (Fig. 2F). In the tail, *dnm2a* expression varied with somite maturation. During somitogenesis *dnm2a* expression was observed in paraxial somitic mesoderm and in the adaxial cells, delineating comb-like structures (Fig. 2A,G,H); at 24 hpf the transcript appeared in newly formed somites in the tail, progressively disappeared from more rostral somites (Fig. 2B and supplementary Fig. 2), and became less intense in the CNS.

To assess whether *dnm2a* expression was related to somite formation, we performed double staining with myf5, a muscle-specific transcription factor that regulates myogenesis^13^ and is expressed in the paraxial mesoderm of somites during early embryogenesis^14^. *Dnm2a* transcript expression only partially overlapped with myf5 expression: in dorsal view flat-mounted embryos, *dnm2a* was expressed in the posterior part of somites and close to the notochord, against a background of uniform myf5 expression in somites (Fig. 2G). Transverse sections showed that *dnm2a* was present in the medio-ventral part of the somite and in adaxial cells, while myf5 was expressed laterally in fast muscle precursors (Fig. 2H)^15^.

### Morpholino-mediated knockdown of *dnm2a* in zebrafish

**Figure 3:**
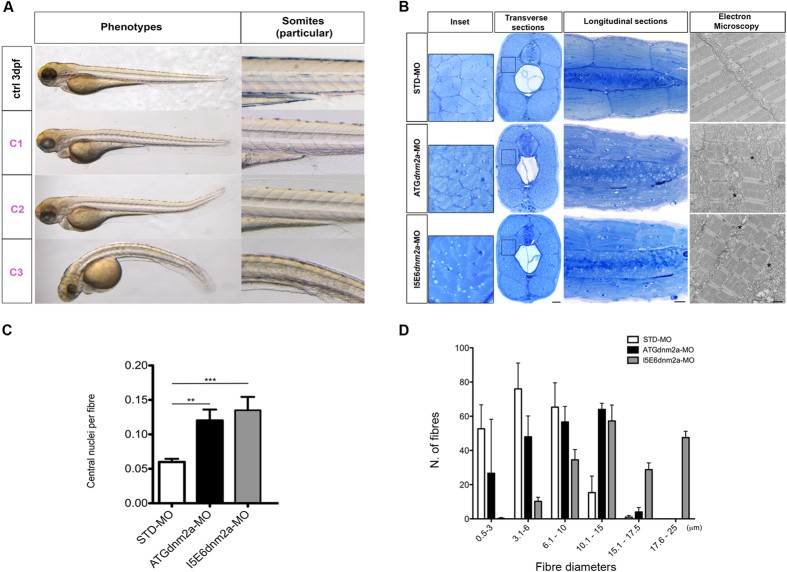
Morphological analysis and quantifications of morpholino-injected embryos. (**A**) Morphological features of STD-MO (ctrl) and morphants, observed under DMR microscope and subdivided into classes according to somite appearance: C1 completely formed, C2 partially disrupted, C3 unformed or totally disrupted somites (morphological features of ATG*dnm2a*-MO and I5E6*dnm2a*-MO-injected embryos are overlapping and representative images are shown). (**B** left) Toluidine blue-stained transverse and longitudinal sections at 4 dpf show evident muscle fibre disorganization in ATG*dnm2a*-MO and I5E6*dnm2a*-MO-injected embryos compared to STD-MO. Scale bar = 20 μm. (**B** right) Electron micrographs of longitudinal sections show myofibrils less regularly arranged, abundant membranous structures, vesicles and tubules (asterisks) in ATG*dnm2a*-MO and I5E6*dnm2a*-MO-injected embryos, compared to STD-MO. Scale bar = 1 μm. (**C**) Quantitation of central nuclei per fibre shows significantly more central nuclei in ATG*dnm2a*-MO and even more in I5E6*dnm2a*-MO-injected embryos than STD-MO. (**D**) Quantitation of fibre diameter indicates that the distribution of fibre diameters is shifted towards larger diameters in ATG*dnm2a*-MO and I5E6*dnm2a*-MO-injected embryos compared to STD-MO. Morphological analysis were performed on 6 embryos for each group (ATG*dnm2a*-MO, I5E6*dnm2a*-MO and STD-MO), chosen randomly from 6 independent injections.

To model the human diseases in zebrafish, we used morpholinos (MO) (Gene Tools Philomath, USA) to block *dnm2a* translation. Specifically we used an antisense oligonucleotide against the start site of the *dnm2a* transcript (ATG*dnm2a*-MO), and an MO to target the splice site between intron 5 and exon 6 (I5E6*dnm2a*-MO) causing a frame-shift and introducing an early down-stream stop codon (Supplementary Fig. 3). We tested both MOs at a range of concentrations (from 0.16 pmol/embryo to 0.96 pmol/embryo) and observed dose-dependent phenotypic classes (Supplementary Fig. 4). In all experiments, MO-injected embryos were compared to embryos at the same developmental stage injected with the same amount of a non-specific standard MO (STD-MO). We observed similar phenotypes when ATG*dnm2a* and I5E6*dnm2a* MOs where injected separately at different concentrations. In order to produce optimal numbers of normal-appearing embryos with a conserved overall somite structure, we injected (based on our preliminary injections at different concentrations) ATG*dnm2a*-MO at a concentration of 0.64 pmol/embryo and I5E6*dnm2a*-MO at a concentration of 0.32 pmol/embryo. At these concentrations, 361 embryos injected with ATG*dnm2a*-MO (49% of total) had normal-appearing morphology with completely formed somites (pertaining therefore to the C1 class, Fig. 3A, particular), 243 (33% of total) had partially disrupted somites (C2 class, Fig. 3A, particular) and 132 (18%) had unformed or totally disrupted somites (C3 class, Fig. 3A, particular); while 342 embryos injected with I5E6*dnm2a*-MO were belonging to the C1 class (48% of total), 292 embryos to the C2 class (41% of total), and 78 embryos to the C3 class (11% of total).

We assessed MO efficiency in C1 embryos by analysing Dnm2a protein levels by Western blot. We found that both morpholinos greatly reduced the intensity of the Dnm2a band (Supplementary Fig. 5) while the BIN1 and actin (internal controls) bands were of normal intensities.

### Morphologically altered phenotype is specifically linked to *dnm2a* gene knockdown

To test the specificity of ATG*dnm2a*-MO and I5E6*dnm2a*-MO, we co-injected low doses of both morpholinos (0.10 pmol/embryo of each), and evaluated morphologic alterations in embryos at 3 dpf.

We first injected separately 0.10 pmol/embryo of ATG*dnm2a*-MO, and found 63 embryos (94% of total) belonging to the C1 class, 3 embryos (5% of total) to the C2 class, and 1 embryo (1%) to the C3 class. By injecting 0.10 pmol/embryo of I5E6*dnm2a*-MO, 57 embryos (77% of total) belonged to the C1 class, 15 embryos (20% of total) to the C2 class, and 3 embryos (3% of total) to the C3 class. When low doses of the ATG*dnm2a*-MO and I5E6*dnm2a*-MO were co-injected in the same embryo, we observed 23 embryos (26% of total) belonging to the C1 class, 41 embryos (47% of total) belonging to the C2 class, and 24 embryos (27% of total) belonging to the C3 class. As control we injected 0.10 pmol/embryo of STD-MO, observing 57 embryos belonging to C1 class (94% of total), 4 embryos belonging to C2 class (6% of total) and none embryo belonging to C3 class (Supplementary Fig. 6).

These results showed that, when combined, the morpholinos could cause severe morphological alterations even at doses that were negligible on their own, confirming their targeting specificity.

### Touch evoked response test in embryos after *dnm2a* knockdown

We performed the touch evoked response test in 3 dpf embryos belonging to the C1 class after injection with either ATG*dnm2a*-MO or I5E6*dnm2a*-MO. This test involves observing an embryo’s swimming behaviour in response to tactile stimulation. In comparison to STD-MO injected embryos, ATG*dnm2a*-MO morphants presented either almost total absence of escape contraction or, more often, they weakly flexed; I5E6*dnm2a*-MO embryos always displayed a weak escape contraction, followed by slow swimming and moving for only a short distance (Supplementary Videos 1–3).

### Morphological abnormalities in muscles after *dnm2a* knockdown

We studied larvae at 4 dpf, when muscle formation was complete, after conclusion of the two myogenic waves^15^,^16^,^17^. Toluidine blue-stained transverse and longitudinal sections showed evident muscle fibre disorganization in MOs-injected embryos compared to STD-MO controls (Fig. 3B). Similarly, longitudinal section electron micrographs revealed altered myofibril organization, abundant membranous structures and prominent vesicles and tubules, in MOs-injected embryo muscle compared to control muscle (Fig. 3B).

Toluidine blue-stained transverse sections (0.4 μm thick), taken from the trunk-tail region had significantly greater numbers of central nuclei per muscle fibres in morphants than controls (ATG*dnm2a*-MO: 0.12 ± 0.02; I5E6*dnm2a*-MO: 0.13 ± 0.04; STD-MO: 0.06 ± 0.004; p = 0.003 and p = 0.0002 respectively) (Fig. 3C). Muscle fibre diameters were also significantly larger than those in control embryos (fibres 10.1 μm or larger were: 28.1% in ATG*dnm2a*-MO vs. 10.7% in STD-MO, and 42.7% in I5E6*dnm2a*-MO vs. 10.7% in STD-MO; p = 0.0006 and p < 0.0001 respectively; Chi Square test). There were also significantly fewer fibres per transverse section in ATG*dnm2a*-MO embryos compared to STD-MO (ATG*dnm2a*-MO: 173.10 ± 5.97 vs. STD-MO: 223.0 ± 14.99; p = 0.009); while in I5E6*dnm2a*-MO embryos the number of fibres per transverse section was not significantly different from STD-MO embryos (I5E6*dnm2a*-MO: 178.5 ± 8.84 vs. STD-MO: 223.0 ± 14.99; p = 0.621) (Fig. 3D).

### Rescue of *dnm2a* knockdown embryos by expression of *dnm2a-v1* and *dnm2a-v2*

In order to demonstrate that the defects observed in *dnm2a* morphants were specifically due to protein reduction induced by the ATG*dnm2a*-MO or I5E6*dnm2a*-MO, we performed rescue experiments in *dnm2a* knocked down embryos. We injected the two *dnm2a* spliced isoforms, the one previously reported, lacking a complete PRD domain, and the one we had found.

**Figure 4:**
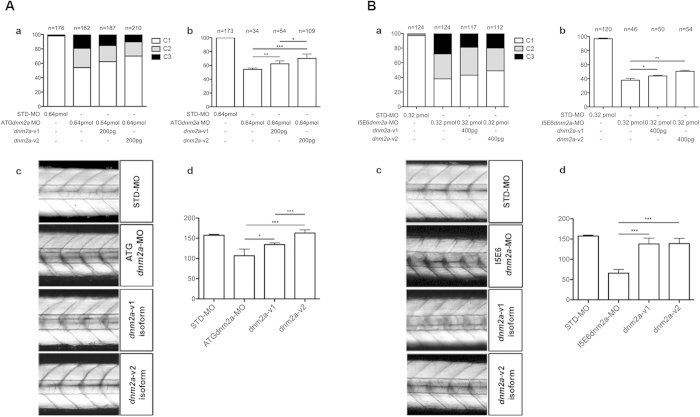
(**A**) Rescue with *dnm2a*-v1 and *dnm2a*-v2 after injection of ATG*dnm2a*-MO. (a) Appearance of embryos at 3 dpf after injection of ATG*dnm2a*-MO and either *dnm2a*-v1 or *dnm2a*-v2 rescue. The proportion of normal-appearing embryos (C1) is greater after rescue with *dnm2a*-v1 and *dnm2a*-v2 than after treatment with ATG*dnm2a*-MO alone. (b) Touch evoked response test results in normal-appearing embryos (C1) identified in experiments performed in (a). The percentage of embryos with normal touch evoked response was significantly greater in touch evoked after rescue with *dnm2a*-v1 and *dnm2a*-v2 than embryos injected with ATG*dnm2a*-MO alone (results obtained from 5 indipendent experiments). (c) Analysis of birefringence at 3 dpf in n = 3 somites after the end of the yolk in 6 independent replicates. Birefringence is evident in the muscle of STD-MO embryos, reduced in somites of ATG*dnm2a*-MO embryos and reverted to almost normal in embryos rescued with *dnm2a*-v1 and *dnm2a*-v2. (c) Graph shows quantitation of birefringence in STD-MO embryos, ATG*dnm2a*-MO embryos, embryos rescued with *dnm2a*-v1 and embryos rescued with *dnm2a*-v2. (**B**) Rescue with *dnm2a*-v1 and *dnm2a*-v2 after injection of I5E6*dnm2a*-MO. (a) Appearance of embryos at 3 dpf after injection of I5E6*dnm2a*-MO and either *dnm2a*-v1 or *dnm2a*-v2 rescue. The proportion of normal-appearing embryos (C1) is greater after rescue with *dnm2a*-v1 and *dnm2a*-v2 than after treatment with I5E6*dnm2a*-MO alone. (b) Touch evoked response test results in normal-appearing embryos (C1) identified in experiments performed in (a). The percentage of embryos with normal touch evoked response was significantly greater in touch evoked after rescue with *dnm2a*-v1 and *dnm2a*-v2 than embryos injected with I5E6*dnm2a*-MO alone (results obtained from 4 independent experiments). (c) Analysis of birefringence at 3 dpf in n = 3 somites after the end of the yolk in 6 independent replicates. Birefringence is evident in the muscle of STD-MO embryos, but significantly reduced in somites of I5E6*dnm2a*-MO embryos and reverted to almost normal in embryos rescued with *dnm2a*-v1 and *dnm2a*-v2. (c) Graph shows quantitation of birefringence of STD-MO embryos, I5E6*dnm2a*-MO embryos, embryos rescued with *dnm2a*-v1 and embryos rescued with *dnm2a*-v2.

For ATG*dnm2a* knockdown rescue, one-cell stage embryos were co-injected with ATG*dnm2a*-MO (0.64 pmol) and either *dnm2a*-v1 mRNA or *dnm2a-*v2 mRNA. At 3 dpf, the proportion of embryos with wild type phenotype was greater and the proportions with partially disrupted somites, and unformed or totally disrupted somites, were smaller with both rescue mRNAs compared to those injected with ATG*dnm2a*-MO alone (Fig. 4A,a).

The touch evoked escape test, performed in embryos with non-altered somites, showed that the percentage of embryos that moved normally increased from 18%, when injected with ATG*dnm2a*-MO, to 27% with injection of *dnm2a-*v1 (p = 0.0456), and to 52% with injection of *dnm2a-*v2 (p < 0.001). Comparison of *dnm2a-*v1 vs. *dnm2a-*v2 injection, showed a significantly greater rescue with the latter (p = 0.0126) (Fig. 4A,b).

To further assess muscle rescue in ATG*dnm2a*-MO zebrafish larvae we performed birefringence analysis^18^ finding that the positions of fibres in somites appeared more regular after injection of *dnm2a-*v1, and more so after injection of *dnm2a-*v2 in comparison to embryos injected with ATG*dnm2a*-MO alone (ATG*dnm2a*-MO vs. *dnm2a-*v1, p = 0.01; ATG*dnm2a*-MO vs *dnm2a-*v2, p = 0.0002). Comparison of *dnm2a-*v1 vs. *dnm2a-*v2 injection showed that the increase in birefringence with the latter was significantly greater (p = 0.0001) (Fig. 4A,c and d).

For I5E6*dnm2a* knockdown rescue, one-cell stage embryos were co-injected with I5E6*dnm2a*-MO (0.32 pmol) and either *dnm2a*-v1 mRNA or *dnm2a-*v2 mRNA (400 pg each). Like for the ATG*dnm2a*-MO, at 3 dpf the proportion of embryos with wild type phenotype was greater, and the proportions of embryos with partially disrupted and unformed or totally disrupted somites were smaller, when rescue mRNAs were used (Fig. 4B,a). The touch evoked escape test, also performed in embryos with non-pathological somites, showed that the percentage of embryos that moved normally increased from 37% to 43% with injection of *dnm2a-*v1 (p = 0.02), and to 49% with injection of *dnm2a-*v2 (p = 0.002) (Fig. 4B,b); while comparison between *dnm2a-*v1 vs. *dnm2a-*v2 rescued embryos failed to show any significant difference (p = 1). Birefringence analysis showed that in embryos rescued with *dnm2a-*v1 or *dnm2a-*v2 there was a significant increase in birefringence compared to I5E6*dnm2a*-MO injected alone embryos (I5E6*dnm2a*-MO vs. *dnm2a-*v1, p < 0.0001; and I5E6*dnm2a*-MO vs. *dnm2a-*v2, p < 0.0001), and that there was no significant difference when comparing *dnm2a-*v1 vs. *dnm2a-*v2 rescued embryos (p = 0.9215) (Fig. 4B,c and d).

### Dose-dependent motility defects in embryos administered mutated human *DNM2* mRNA

We injected one-cell embryos with either *DNM2* mRNA containing the R522H mutation that causes CNM, *DNM2* mRNA containing the G537C mutation that causes CMT, or wild-type *DNM2* mRNA. The R522H mutation was obtained from a patient’s cDNA; the G537C mutation was obtained by mutagenesis. We assessed embryos after injecting increasing quantities (from 50 pg/embryo to 600 pg/embryo) of WT and mutated mRNAs, and found that 24 hpf mortality was dose-dependent (Data not shown). The frequency of surviving embryos was higher at every tested concentration in WT mRNA injected embryos compared to mutated mRNAs injected embryos. In order to have greater numbers of embryos with less severe movement defects suitable for our analyses, we chose to inject 200 pg of mRNA (Supplementary Fig. 7).

We found that movements in response to touch stimulus in surviving embryos at 48 hpf were altered, and alterations depended on the type of mRNA injected. After injection of 200 pg CNM mRNA, 75% (n = 128) of surviving embryos had altered touch responses; after injection of 200 pg CMT mRNA 73% (n = 132) of embryos had altered touch responses; after injection of 200 pg wild-type mRNA, only 12.2% (n = 130) had altered responses. The type of movement alterations observed in embryos injected with CNM mRNA, consisted in weak contraction that produced a circular instead of a linear escape trajectory. In embryos injected with CMT mRNA motion was severely impaired and characterized by instantaneous overturning of the animal without escape (Supplementary videos 4–6).

### Morphological studies on embryos administered mutated human *DNM2* mRNA

To determine morphological correlates of motility defects, we used monoclonal antibodies znp-1 and zn-5, and α-bungarotoxin (α-btx) staining to detect primary motor axons, secondary motor axon projections, and acetylcholine receptors (AChRs) at neuromuscular junctions, respectively, in 48 hpf embryos^19^,^20^,^21^.

**Figure 5:**
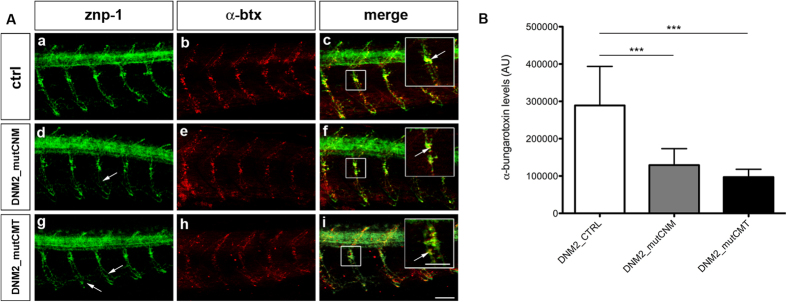
Primary motor neurons (znp-1) and AChRs (α-btx) in human mRNAs injected embryos. (**A**) Co-localization of markers for primary motor neurons (znp-1) and AChRs (α-btx) in 5 spinal hemisegments and somites, in 48 hpf zebrafish embryos. The images are representative of those found in n = 10 embryos for each condition, during 3 distinct experiments. In DNM2_mutCNM*-*injected embryos (d,e) and DNM2_mutCMT*-*injected embryos (g,h) primary motor axons migrate normally along the common path (compare a with d and g) with slight pathfinding and shape defects after the choice point (arrows). The merge images suggest that DNM2_mutCNM*-* and DNM2_mutCMT*-*injected embryos present fewer α-btx-positive spots than DNM2-controls, with the co-localization signal is reduced in intensity (f,i), compared to control (c), even though znp-1 and α-btx signals co-localize correctly (arrowhead in the enlargement, scale bar = 25 μm). Scale bar = 20 μm. (**B**) Quantification of α-btx-positive spots in n = 10 complete embryos for each condition. DNM2_mutCNM and DNM2_mutCMT*-* injected embryos present fewer α-btx-positive spots than controls. Error bars are SEMs. Scale bar = 20 μm.

By confocal microscopy, primary motor axons in embryos administered with wild-type mRNA showed normal migration and extension along common axonal trajectories, and the overlap between znp-1 and α-btx, was similar to that in non-injected embryos. In embryos injected with CMT or CNM mRNA, primary motor axons migrated normally along the common path, but minor defects in pathfinding and in shape after the choice point were evident (Fig. 5A). Furthermore, a reduced presence of α-btx-positive spots was detected in both groups of embryos (Fig. 5A). Densitometric quantitation confirmed that the α-btx-positive spots were significantly reduced (p < 0.0001 for both CNM and CMT; Chi Square test) compared to controls (Fig. 5B).

**Figure 6:**
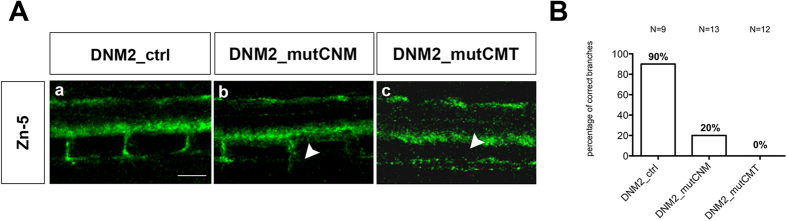
Secondary motor neurons (zn-5) in embryos injected with human mRNAs. (**A**)Visualization of secondary motor neurons by zn-5 monoclonal antibody-labelling of 48 hpf zebrafish embryos. By 48 hpf secondary motor neurons have completed their migration along the common path, and axons of the ventral nerve extend to the ventral myotome in both wild-type embryos (not shown) and those injected with wild-type DNM2 mRNA (DNM2_ctrl) (a). However in DNM2_mutCNM embryos, secondary motor neuron axons appear to exit the spinal cord and migrate to the periphery, but branching is rare (b). By contrast, in embryos expressing DNM2_mutCMT secondary motor axons are not observed (c). Scale bar = 10 μm. The images are representative of those found in average12 embryos for each condition, during 3 independent experiments. (**B**) Graph shows quantitation of branchings.

By confocal microscopy, secondary motor axons, in embryos injected with the wild-type *DNM2* mRNA, migrated normally and completely along the common path, in a similar way to neuronal migration in non-injected embryos (data not shown). However, defects were evident in embryos injected with CNM or CMT mRNAs (Fig. 6A). In particular, in embryos expressing CNM mRNA, although axons appeared to correctly exit the spinal cord and migrate to the periphery, branching was rare (Fig. 6A,b). In embryos expressing CMT mRNA, secondary motor axons were completely absent (Fig. 6A,c).

**Figure 7:**
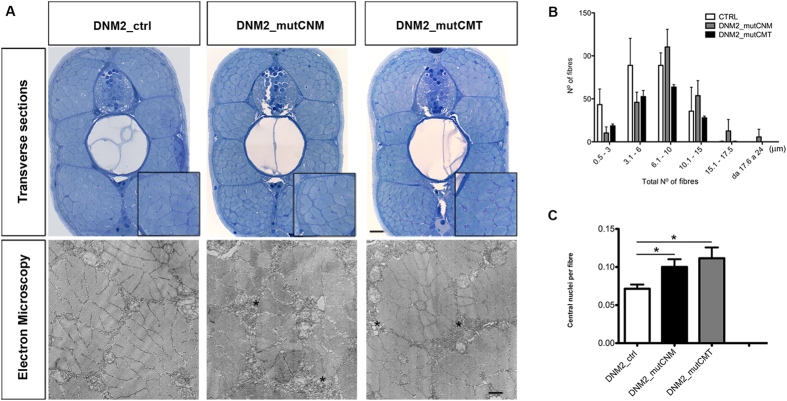
Morphological analysis and quantifications of human mRNAs injected embryos. (**A**) Toluidine blue-stained transverse sections and electron micrographs at 4 dpf of DNM2_mutCNM and DNM2_mutCMT -injected embryos in comparison to control (DNM2_ctrl). In Toluidine blue-stained sections both CNM and CMT injected embryos muscle tissue has more disordered morphology than control embryos, with fibres of variable size, frequent lobular appearance and more space surrounding fibres. Scale bar = 10 μm. Electron micrographs show greater vesicular decoration in spaces surrounding fibres (asterisks) and loose morphology. Scale bar = 0.5 μm. (**B**) Quantitation of fibre diameter indicates that the distribution of fibre diameter is shifted towards larger diameter in CNM- and CMT-injected embryos compared to control (in DNM2_mutCNM and DNM2_mutCMT 30.2% and 17.3% respectively of fibre diameters were 10.1 μm or greater, compared to 13.3%in control p < 0.0001). (**C**) Quantitation of central nuclei per fibre shows significantly more central nuclei in both CNM- and CMT-injected embryos than control. Morphological analysis were performed on n = 6 embryos for DNM2_mutCNM, DNM2_mutCMT and DNM2_ctrl each, chosen randomly from 4 different injections.

We examined muscles from the trunk-tail region in larvae at 4 dpf injected with CNM, CMT or control mRNAs. Toluidine blue-stained transverse sections (0.4 μm thick) showed that in both CNM- and CMT-injected embryos, muscle tissue had a more disordered morphology than controls, with fibres of variable size, frequent lobular appearance, and more space surrounding fibres (Fig. 7A). Electron micrographs appeared to show greater vesicular decoration in spaces surrounding fibres and less orderly morphology compared to control embryos (Fig. 7A).

Numbers of central nuclei per total number of fibres were significantly greater in CNM- (0.10 ± 0.01; p = 0.030) and CMT- (0.11 ± 0.01; p = 0.022) injected embryos than controls (0.07 ± 0.005). Total numbers of fibres per section were not significantly lower after CNM injection (242.9 ± 12.91; p = 0.103), but were significantly fewer after CMT injection (202.1 ± 19.47; p = 0.008) relative to control (273.4 ± 11.57). Fibre diameter (Fig. 7B) was also significantly greater in CNM and CMT embryos than control (fibre diameters of 10.1 μm or greater were 30.2% and 17.3% in CNM and CMT, respectively, compared to 13.3% in controls, p < 0.0001).

## Discussion

Recent years have seen substantial advances in our understanding of centronuclear myopathy and Charcot Marie Tooth dominant intermediate type B diseases; however little is known about overlapping aspects of these two pathologies.

For this reason, the rapid zebrafish *in vivo* model appeared as a useful analytical tool to investigate the effects of *dynamin-2* mutations on the neuromuscular system in CNM and CMT.

To be able to fully understand *dynamin 2*-related disorders in zebrafish, we first silenced *dnm2a* using two different morpholinos, one directed against the start site of the *dnm2a* transcript (ATG*dnm2a* knockdown), and one that targeted the splice site between intron 5 and exon 6 (I5E6*dnm2a* knockdown). ATG*dnm2a* knockdown resulted in severe abnormalities of the muscular system in zebrafish, similar to those documented previously^10^, while I5E6*dnm2a* knockdown induced different motility defects, although morphological features were similarly altered in both ATG*dnm2a* and I5E6*dnm2a* knockdown.

We then introduced in zebrafish embryos *DNM2* transcripts containing mutations that cause CNM or CMT in humans. This is a most novel aspect of our study that, taking advantage of the high identity between the human and zebrafish dynamin-2 proteins, allowed us to mimic the patient situation in which both the wild-type and the pathological allele are present. Since many human dynaminopathies are autosomal dominant and ascribed to gain of function of the aberrant protein^6^, our model promises to be useful for investigating mechanisms of human dynaminopathies. The introduction of *DNM2* transcripts containing mutations that cause CNM or CMT in humans, resulted indeed in severe neuromuscular abnormalities in zebrafish embryos similar to some of those observed in the human diseases.

Building upon previous work^10^, novel phenotypes were revealed by comparing two different mutations, one causing CNM, and the other causing CMT, both positioned very close in the PH domain of the DNM2 protein. Human transcripts with mutations in the PH domain give rise to a variety of disease phenotypes in humans, from late onset CNM^22^ to severe CMT^8^. Although some overlap of CNM and CMT pathologies has been observed in patients with PH domain mutations, no known *DNM2* mutation causes both CNM and CMT^23^,^24^,^25^. Specifically, we used mRNA containing the R522H mutation, present in three of our CNM patient cohort with variably severe phenotypes. We also used an mRNA containing the G537C mutation, known to cause CMTDIB neuropathy^4^,^26^,^27^.

We found that both mutated transcripts produced morphological defects that primarily involved secondary, rather than primary motor neurons.

Similarities between zebrafish secondary motor neurons and those of birds and mammals are greater than similarities between the corresponding primary motor neurons^28^,^29^. Zebrafish secondary motor neurons are in fact similar to human α motor neurons^28^,^30^ and thus zebrafish promise to be a useful model for elucidating mechanisms of human lower motor neuron disease.

At 48 hpf, zebrafish motor neurons have extended branches to form synapses with laterally located muscle fibres, turned laterally at the edge of the myotome, and grown along and innervated myosepta. Between 48 and 72 hpf, secondary motor axons extend into the myotome following pathways pioneered by the primary motor axons^31^,^32^,^33^,^34^. Introduction of CNM mRNA (containing the R522H mutation) produced mild defects in zebrafish primary motor neuron morphology, and severe and obvious defects in secondary motor neuron morphology, resulting in incorrect branching of the latter. Introduction of CMTDIB mRNA (containing the G537C mutation) produced defects in primary motor neurons similar to those induced by CNM mRNA, while secondary motor neurons were absent.

The touch response test also confirmed that defects in CMT mRNA-injected embryos were more severe compared to those injected with CNM mRNA. Furthermore, the significantly lower numbers of fibres per section than controls, found in CMT, but not in CNM mRNA injected embryos, are likely to result from more severe defects in innervation/maturation of muscle in the CMT model. As in human motor neuron diseases, denervation or lack of innervation are likely to cause fibre atrophy, while persisting fibres are likely to become hypertrophic as compensatory effect.

Based on our findings, therefore we can conclude that the zebrafish CNM and CMT models show some of the characteristics of human centronuclear myopathy and Charcot Marie Tooth neuropathy dominant intermediate type B, respectively.

However, these two models also showed some closely similar defects such as the increase in central nuclei per total number of fibres, and the increase in fibre diameters, compared to controls. To this regard we note that clinical overlap with CMT has been reported for some CNM patients presenting with a mild peripheral nerve involvement^35^, and that EMG results in some of the patients described by Bohm *et al.*^25^ and Echaniz-Laguna *et al.*^36^ were suggestive of neuropathy. Only two patients (mother and son) with the G537C mutation (reported in the article as G533C, following previous annotation) causing CMT have been reported^37^. The neuropathy in these patients was characterized by mild-to moderate impairment with scarce evolution in middle age, upper limbs relatively preserved, and asymmetric involvement of calves affecting muscles of both anterior and posterior compartments.

Primary myopathic features in CMT patients with *DNM2* mutations have never been reported, including in the two patients with the G537C mutation. Since muscle biopsy is not usually performed in these patients, presence of central nuclei in muscle fibres cannot be completely ruled out.

With the present study we emphasize that morphometric techniques, normally applied to study histopathology in human muscle, can be usefully employed to evaluate muscle histopathology in zebrafish, underlining the versatility of this animal model.

Interestingly, working on the zebrafish dynamin-2 gene led us to identify a new *dnm2* transcript (*dnm2a-*v2). This is much closer – in terms of length and sequence, to the normal human *DNM2* transcript – than the previously identified transcript (renamed *dnm2a-*v1). We have shown that *dnm2a*-v2 is not present before the epiboly stage, while *dnm2a*-v1 is present from the earliest developmental stages^11^,^12^. The two isoforms could be both developmentally regulated and produced by the zygote; alternatively the long transcript could have a zygotic origin, while the short one could be provided maternally^12^. Computational analysis indicated the presence of a longer *dnm2a* isoform with a complete PRD domain (present partially in the short isoform). To confirm this idea with experimental data, we performed rescue analysis on morphants with both *dnm2a* isoforms. The muscular abnormalities were rescued by the introduction of either *dnm2a-*v1 or *dnm2a-*v2 transcripts, but the injection of *dnm2a*-v2 produced a greater number of rescued animals (Fig. 4B).

To conclude, our data confirm that the zebrafish *dnm2a* knockdown is a valuable model for dynaminopathies and, most importantly, demonstrate that overexpression of human *DNM2* mRNAs, containing different disease-related mutations, cause a continuum of pathological features similarly to what observed in human centronuclear myopathies and neuropathies. This characteristic allowed us to mimic the patient situation in which both the wild-type and the pathological allele are present.

Therefore, zebrafish is a rapid, low-cost and powerful screening tool to address the study of mutational effects *in vivo* to understand the mechanisms underlying *DNM2*-related diseases, and, possibly, to accelerate the identification of new therapeutic targets.

## Materials and Methods

### Animal care

The studies on Zebrafish were approved by the animal ethics committee of the University of Milan and carried out at the University facility. Animals were always injected according to the principles of Good Animal Practice as defined by Italian animal welfare regulations. The experiments were performed on zebrafish (AB strain) embryos and larvae between 1 and 4 days post fertilization (dpf).

### 3′RACE

To verify bioinformatics results we performed 3′RACE experiments, using the following primers:


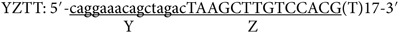


Y: 5′-CAGGAAACAGCTATGAC-3′

Z: 5′-TGACTAAGCTTGTCGACG-3′

X0dnm2a: 5′-GCATCTCATGATCAACAGCG-3′

X1dnm2a: 5′-GACGAGATGCTGAGGATG-3′

X2dnm2a: 5′-AGCACCAGCACCATTTCC-3′

X3dnm2a: 5′-CCAGGATCCTTTCAGCGC-3′

R: 5′-GTCCTCGGCGTGCTTTGGC-3′.

First amplification was carried out with primers X0 and Z with 1 μl of X0/Y PCR mixture as template. The conditions were 95 °C for 2 min, 52 °C for 10 sec, and 72 °C for 1 min (1 cycle) and 95 °C for 5 sec, 52 °C for 10 sec, and 72 °C for 1 min (33 cycles). Seminested PCR with primers Z1.2N and Z were necessary to obtain sufficient specific cDNA for cloning and sequencing.

### RT-PCR

Total RNA was extracted from zebrafish embryos at different stages, or from human muscle biopsies using TRI Reagent (MRC, Cincinnati, OH, USA). First-strand cDNA synthesis reaction from total RNA was catalyzed by Transcriptor First Strand cDNA Synthesis Kit (Roche Diagnostic, Penzberg, Germany). cDNA was amplified with specific primers using Phusion High-Fidelity polymerase (Finnzymes, Thermo Fisher Scientific, Waltham, MA, USA). The PCR products were purified using Illustra ExoProStar (GE Healthcare, Life Sciences, WI, USA) and sequenced directly with BigDye Terminator v1.1 Cycle Sequencing Kit (Applied Biosystems, Life Technologies, Carlsbad, CA, USA). Sequences were analyzed on an ABI Prism 3100 Genetic Analyzer (Applied Biosystems, Life Technologies, Carlsbad, CA, USA).

The primers used to clone the cDNA in the vector were:

*dnm2a*-v1: 5′-TACAACACATGCGCGTCTTC-3′ (Forward) and 5′-CAACGACAGCTGGATACCTG-3′ (Reverse); *dnm2a*-v2: 5′-CCTCTGATCACAGCACGTATT-3′ (Forward) and 5′-TAAGTGTCCTCTGACCAGCG-3′ (Reverse); human-*DNM2*: 5′-GGGAGCAACGGCTACAGAC-3′ (Forward) and 5′-CCCAGACCACTGAAGCTCCT-3′ (Reverse).

The primers for RT-PCR analysis of v1 and v2 isoform expression were: *dnm2a-*v1: 5′-CGTATTACAACACATGCGCG-3′ (Forward) and 5′-catgcgtgccaaagaatagga-3′ (Reverse), with the reverse primer designed on the 3′UTR, which is different for the v1 and the v2 isoform; *dnm2a*-v2: 5′-CCTCTGATCACAGCACGTATT-3′ (Forward) and 5′-TAAGTGTCCTCTGACCAGCG-3′ (Reverse).

### *In situ* hybridization

Whole-mount RNA *in situ* hybridization was carried out using probes made by *in vitro* transcription with T7 or SP6 RNA polymerase (Promega Corporation, Madison, WI, USA). Templates were generated by PCR using the following primers: 5′-GTCTAATCCTTGCCGTCACC-3′ (Forward, SP6), and 5′-TAAGAACTTCCGCCCAGATG-3′ (Reverse, T7), common to the v1 and v2 isoforms. PCR was performed on cDNA from 1 dpf wild-type embryos. The probe and template sequences were verified.

For histological analysis, stained embryos were fixed in 4% paraformaldehyde, dehydrated, wax embedded, sectioned (8 μm) with microtome (Leitz 1516) and stained with eosin. Images were taken with an Olympus BH2 microscope, equipped with a Leica DFC 320 digital camera and IM50 software (Leica).

### Morpholino injections

For *dnm2a* knockdown, the following morpholinos were designed and purchased, along with standard control morpholino, from Gene Tools (Gene Tools, Philomath, OR, USA): 5′-ACctacgacaagggaaaaatcacat-3′ (I5E6*dnm2a*-MO) and 5′- ctcgggttactttcaagtgttcag-3′ (ATG*dnm*2a-MO).

Fertilized eggs were collected after timed mating of adult zebrafish and injected at the 1 – 2-cell stage using an Eppendorf transferman nk2 micromanipulator (Eppendorf). Embryos were injected with either I5E6*dnm*2a-MO (0.32 pmol/embryo) or ATG*dnm*2a-MO (0.64 pmol/embryo) or morpholino (to verify absence of morpholino-mediated toxicity) in a volume of 4 nl.

For rescue experiments, embryos were co-injected (total volume 4 nl) with ATG*dnm2a*-MO morpholino plus either v1-isoform_mRNA (200pg) or v2-isoform_mRNA (200pg) and with I5E6*dnm2a*-MO morpholino plus either v1-isoform_mRNA (400pg) or v2-isoform_mRNA (400pg).

Morpholinos were diluted in Danieau solution^38^. Rhodamine dextran (Molecular Probes, Life Technologies, Carlsbad, CA, USA) was usually co-injected as tracer to enable monitoring with a Leica MZ FLIII epifluorescence microscope equipped with a Leica DCF 480 digital camera and IM50 software (Leica).

After injection, embryos were allowed to develop in fish water at 28 °C up to the stage of interest.

### Western Blot

Dechorionated embryos (minimum 20 per experiment) were solubilized in RIPA buffer (Radio-Immunoprecipitation Assay buffer) plus protease inhibitor and phenylmethylsulfonyl fluoride,100X (PMSF). Samples were boiled for 10 min at 95^o^ or sonicated. Thirty μg of protein samples were electrophoresed on 10% SDS-PAGE and transferred to nitrocellulose membranes (Bio-Rad Laboratories, Hercules, CA, USA) following standard procedures. The membranes were blocked with 5% nonfat dry milk in TBS, pH 7.5, containing 0.1% Tween 20 (TBST) for 1 h at room temperature and subsequently incubated with the primary antibodies anti-DNM2 (1:50, polyclonal, Santa Cruz Biotecnology, Santa Cruz, CA, USA), anti-BIN1 (1:500, polyclonal, Bethyl Laboratories, Montgomery, USA) and anti-acetylated α-tubulin (1:3000, polyclonal, Sigma-Aldrich, Saint Louis, MO, USA), the latters used as internal control, followed by biotinylated goat anti-rabbit, ABC-Kit Complex (DAKO, Agilent Technologies, Santa Clara, CA, USA), and ECL detection (Bio-Rad Laboratories, Hercules, CA, USA).

### Whole-mount immunohistochemistry and AChR labelling

All antibodies were diluted in blocking solution (5% w/v bovine serum albumin [BSA] in phosphate buffered saline [PBS] with 0.1% Tween20). After overnight fixation in 4% paraformaldehyde at 4 °C, the embryos were washed 5 times for 5 min in 0.1% PBS-Tween, rinsed in water, permeabilized for 15 min in cold acetone, rinsed in water, washed 5 times for 5 min in 0.1% PBS-Tween, incubated in NH_4_Cl solution for 30 min at room temperature, blocked for 3 h at room temperature in blocking solution and, finally, incubated overnight at 4 °C with primary antibody. The next day, the embryos were washed 5 times for 10 min in 0.1% PBS-Tween, then every 2–3 h with blocking solution at room temperature on an orbital shaker until evening, when secondary antibody was applied overnight at 4 °C, followed by washings (3 times for 5 min) in 0.1% PBS-Tween at room temperature. Primary antibodies were: anti-Znp-1 1:200 (mouse anti-syt2b, anti-synaptotagmin 2) or anti-Zn-5 1:200 (mouse anti-alcama, activated leucocyte cell adhesion molecule A), both from Zebra International Resource Center (OR, USA); the secondary antibodies were: Alexa 488-conjugated goat anti-mouse IgG or Alexa 546-conjugated goat anti-rabbit IgG, (Invitrogen Life Technologies, Carlsbad, CA, USA) both diluted 1:200.

Alpha-bungarotoxin (α-btx) labeling was performed as described elsewhere^39^. Briefly, after incubation with primary and secondary antibody, embryos were incubated for 30 min at room temperature in 10 g/ml Alexa 547-conjugated α-btx (Sigma-Aldrich, Saint Louis, MO, USA), diluted in BSA-PBS-Triton (5% normal bovine serum, and 1% Tween in PBS-Triton), washed in PBS-Triton, and mounted in Mowiol.

Confocal microscopy was performed on a Leica TCS SP2 AOBS microscope, equipped with an argon laser (Leica).

### Muscle structure and ultrastructure

Zebrafish at 4 dpf were fixed 2 h in 2.5% glutaraldehyde-sodium phosphate buffer, pH 7.4; left in buffer overnight at 4 °C. The embryos were post-fixed in phosphate-buffered 2% OsO_4_, dehydrated in graded ethanol, and embedded in epoxy resins (Electron Microscopy Sciences, Hatfield, Pennsylvania). Semi-thin sections (0.4 μm thick) were examined by light microscopy after staining with toluidine blue. Ultrathin sections of zebrafish tails were collected onto grids, stained with uranyl acetate and lead citrate, and examined with a FEI electron microscope.

### Quantitation

To quantify the α-bungarotoxin (α-btx) level, the red signal area (expressed as arbitrary units that corresponded to number of pixels) was calculated on each confocal z-stack by means of Fiji software version 2.0 ( http://rsb.info.nih.gov/nih-image/) on micrographs taken at same exposure conditions^40^. Briefly, fields occupied by the entire tail of the animal were photographed from each z-stack and digitalized. Using the software, a threshold was applied to the photographs to obtain red and black images with areas positive for α-btx in red and negative areas in black. The area positive for α-btx was calculated as a percentage of the entire image on each confocal z-stack, and the mean percentage calculated. The same procedure was repeated for each group of animals, and the mean percentage calculated.

Muscle fibres and central nuclei were counted on micrographs of toluidine blue-stained semithin sections reproducing the whole cross-sectional area of the fish in the region immediately after the yolk, taken at 63X under a Zeiss Axioplan2 microscope. Determination of muscle fibre diameters was obtained using Microscope Software AxioVision Release 4.8.2 (Zeiss, Oberkochen, Germany).

### Human cDNA

Total RNA was extracted from skeletal muscle using TRI Reagent (MRC, Cincinnati, OH, USA). First-strand cDNA synthesis reaction from total RNA was catalyzed by Transcriptor First Strand cDNA Synthesis Kit (Roche Diagnostic, Penzberg, Germany). cDNA was amplified with specific primers using Phusion High-Fidelity polymerase (Finnzymes, Thermo Fisher Scientific, Waltham, MA, USA). The PCR products were purified and sequenced directly with BigDye Terminator v1.1 Cycle Sequencing Kit (Applied Biosystems, Life Technologies, Carlsbad, CA, USA). Sequences were analyzed on an ABI Prism 3100 Genetic Analyzer (Applied Biosystems, Life Technologies, Carlsbad, CA, USA). Results were compared to the human cDNA *DNM2* database sequence NM_001005360.1.

### Mutagenesis

Mutagenesis of wild-type human *DNM2* was performed using the QuikChange II XL Site-Directed Mutagenesis Kit (Agilent Technologies, Santa Clara, CA, USA). To introduce the CMTD1B mutation (c.1610G > T) the following primers were designed: 5′-GCCTGATGAAAGGCGTCTCCAAGGAGTACTGG-3′ and 5′-CCAGTACTCCTTGGAGACGCCTTTCATCAGGC-3′. After the reaction, sequences were cloned by transforming XL1-Blue Competent Cells from STRATAGENE (Agilent Technologies, Santa Clara, CA, USA). All sequences were confirmed using an ABI Prism 3130XL Genetic Analyzer. For RNA injection, plasmids were linearized with NotI and transcribed using the SP6 mMessage Machine kit (Ambion, Life Technologies, Carlsbad, CA, USA).

### Plasmid construction

The cDNAs of full-length human *DNM2* and zebrafish *dnm*2a were amplified by RT-PCR using total RNA extracted from human muscle or whole zebrafish embryos. The primer sequences used were 5′- TCCATCGATGGGAGCAACGGCTACAGAC -3′ (sense primer plus ClaI sequence) and 5′- AGCTCTAGACCCAGACCACTGAAGCTCCT -3′ (antisense primer plus XbaI sequence) for Human cDNA, and 5′-GAATTCGAGAGACGGCCAGAAACCAT-3′ (sense primer in common for v1 and v2 isoform plus EcoRI sequence), 5′-CTCGAGCAGGTATCCAGCTGTCGTT-3′ (antisense primer for v1 isoform plus XhoI sequence) and 5′-CTCGAGCGCTGGTCAGAGGACACTTA-3′ (antisense primer for v2 isoform plus XbaI sequence) for zebrafish *dnm2a* cDNA. The amplified fragments were inserted into pCS2+ vector (Clontech, Takara Bio Inc., Otsu, Shiga, Japan). After digestion of pCS2+ vector with appropriate restriction enzymes, isolated cDNA of *DNM2* or *dnm2a* was cloned into the pCS2+ vector (Clontech, Takara Bio, Otsu, Shiga, Japan). The following constructs were established: pCS2+ -*DNM2*, pCS2+ -*dnm2a*-v1 isoform, pCS2+ -*dnm2a*-v2 isoform. The cells used were Library DH5-alpha (Invitrogen Life Technologies, Carlsbad, CA, USA). Enzymes used were: ClaI and XbaI (New England biolabs, Ipswich, MA), and EcoRI and XhoI (Promega Corporation, Madison, WI, USA).

### RNA injection of zebrafish embryos

Zebrafish embryos were injected as described^41^. Briefly, fertilized eggs were injected at the one- to two-cell stage using a Microinjector (Leica). Embryos were injected with Human *DNM2* mRNA (200 pg and 400 pg) or zebrafish *dnm2a* mRNA (200 pg and 400 pg) in a 4 nL volume. Plasmids were linearized with NotI enzyme (New England Biolabs, Ipswich, MA, USA).

### Screening for embryonic motility

At 3 dpf embryos were subjected to a tactile stimulus. Using a needle, a gentle stimulus was applied at the tail of the larvae and its reaction observed. Wild-type larvae at this stage of development have a normal activity. Upon application of the tactile stimulus they swim away from the source of the stimulus^42^.

### Birefringence

Zebrafish at 3 dpf were observed after anesthesia using 0.016% tricaine (Ethyl 3-aminobenzoate methanesulfonate salt (Sigma-Aldrich, Saint Louis, MO, USA) in fish water as described previously^43^. For quantitation, the area occupied by 3 somites positioned immediately after the end of the yolk was selected and the average grey value of the pixels in this area measured by the software Fiji. This procedure was repeated three times and the average calculated for each embryo. Muscle birefringence was analysed for 6 embryos in each group.

### Statistical Analysis

Results were expressed as means ± standard deviation. Differences between fibre diameters and between central nuclei (ATG*dnm2a*-MO and I5E6*dnm2a*-MO vs STD-MO and DNM2_mutCNM and DNM2_mutCMT vs DNM2_ctrl) were assessed using the Chi Square test, and differences between groups in rescue experiments (ATG*dnm2a*-MO, I5E6*dnm2a*-MO*, dnm2a-*v1 isoform, *dnm2a-*v2 isoform and STD-MO), were assessed by the two-tailed Student T test or one-way ANOVA with post-hoc Dunnet test, where appropriate. Differences between DNM2_CMT or DNM2_CNM and DNM2_CTRL were assessed by two-tailed Student t-test. P values were considered significant: *p ≤ 0.05, **p ≤ 0.01; ***p ≤ 0.001.

## Additional Information

**How to cite this article**: Bragato, C. *et al.* Zebrafish as A Model to Investigate Dynamin 2-Related Diseases. *Sci. Rep.* **6**, 20466; doi: 10.1038/srep20466 (2016).

## Supplementary Material

Supplementary Information

Supplementary Video 1

Supplementary Video 2

Supplementary Video 3

Supplementary Video 4

Supplementary Video 5

Supplementary Video 6
