# Supplementary material for: Zebrafish as a Model to Investigate Dynamin 2-Related Diseases
Source: Sci Rep. 2016 Feb 4;6:20466. doi: 10.1038/srep20466 (PMC4740890; doi:10.1038/srep20466)
Supplement: Supplementary Information [file srep20466-s1.pdf]

ZEBRAFISH AS A MODEL TO INVESTIGATE DYNAMIN 2-RELATED DISEASES,

Cinzia Bragato, Germano Gaudenzi, Flavia Blasevich, Giulio Pavesi, Lorenzo Maggi, Michele Giunta, Franco Cotelli & Marina Mora.

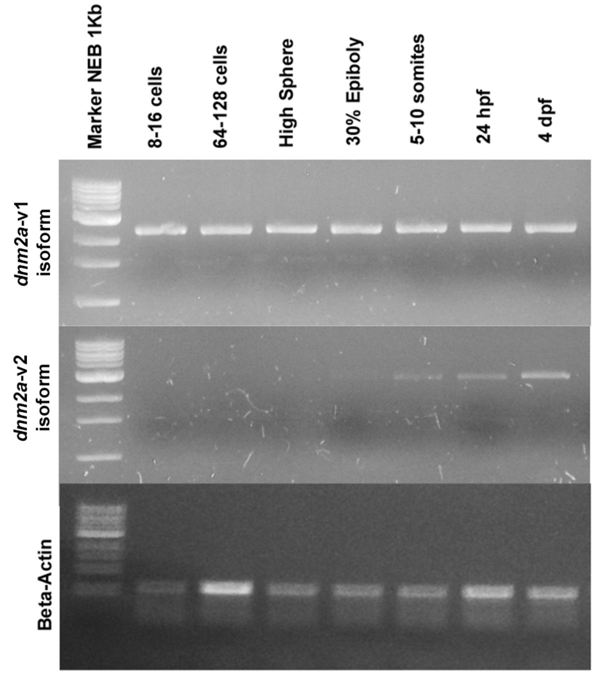

Supplementary Fig. 1. PCR shows the presence of *dnm2a*-v1 transcript from the earliest stages, while *dnm2a*-v2 is not present before the epiboly stage.

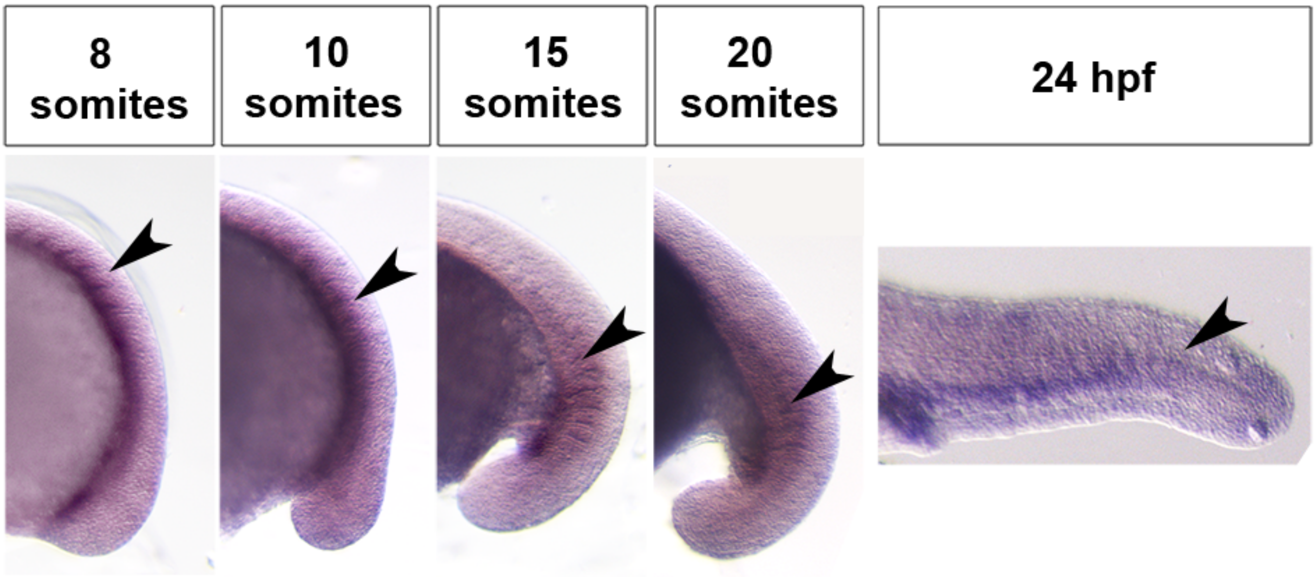

Supplementary Fig. 2. WISH shows the *dnm2a* expression pattern during somitogenesis. In the tail, *dnm2a* expression varied within somite maturation. The *dnm2a* signal wad detected in newly formed somites, and progressively disappeared from more rostral ones.

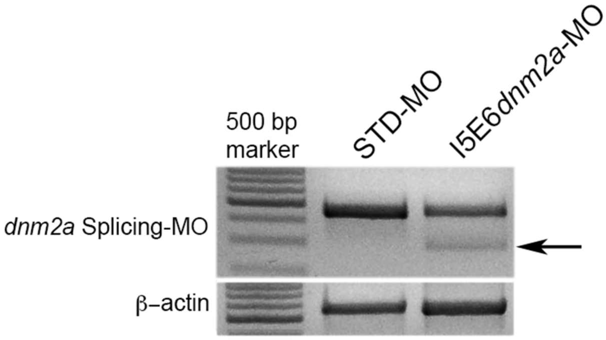

Supplementary Fig. 3. Splice targeting morpholino was designed against intron 5 - exon 6 within the *dnm2a* gene. Efficacy of the splice site targeting morpholino in I5E6*dnm2a*-MO injected embryos was verified using RT-PCR. Embryos were injected with a scrambled control morpholino (STD-MO; 0.32 pmol/embryo) and I5E6*dnm2a*-MO (0.32 pmol/embryo). Black arrow indicate the alternative splice product induced by I5E6*dnm2a*-MO injection. β-actin is the internal control.

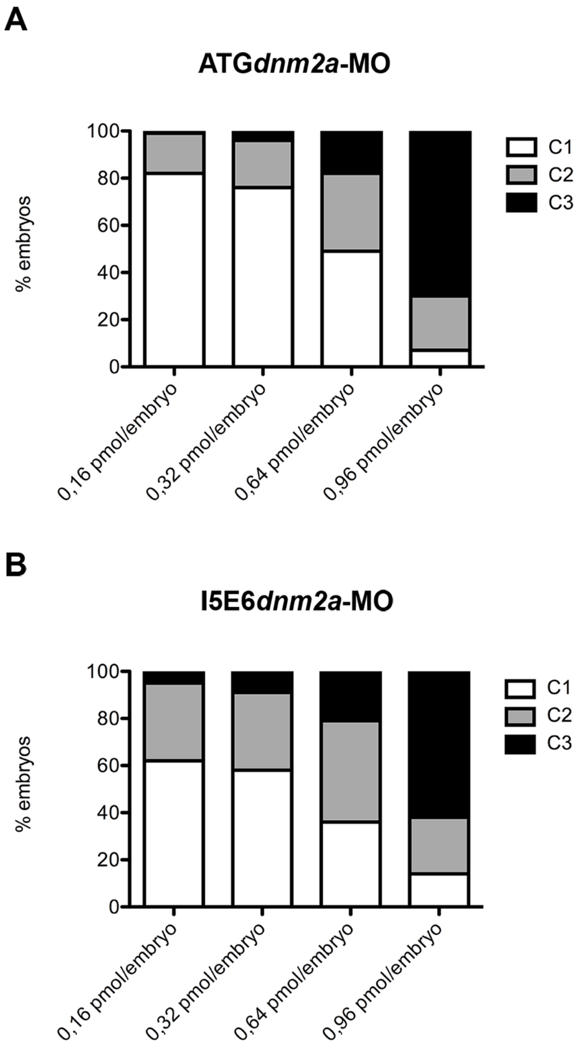

Supplementary Fig. 4. Observed dose-dependent after testing MO at range of concentrations from 0.16 pmol/embryo to 0,96 pmol/embryo. 0.64 pmol/embryo is the chosen concentration for ATG*dnm2a*-MO (n = 736 embryos in 5 independent experiments), and 0.32 pmol/embryo is the chosen concentration for I5E6*dnm2a*-MO (n =

712 embryos in 5 independent experiments). Graph shows the percentage of embryos divided into classes according to morphology.

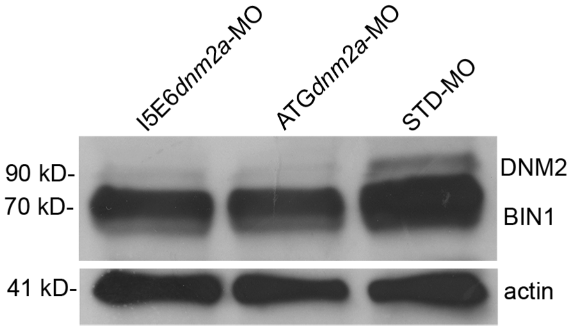

Supplementary Fig. 5. Western blot shows reduced Dnm2a (90kD) expression levels in ATG*dnm2a*-MO and I5E6*dnm2a*-MO-injected embryos. Actin (41kD) and BIN1 (70kD) are internal controls.

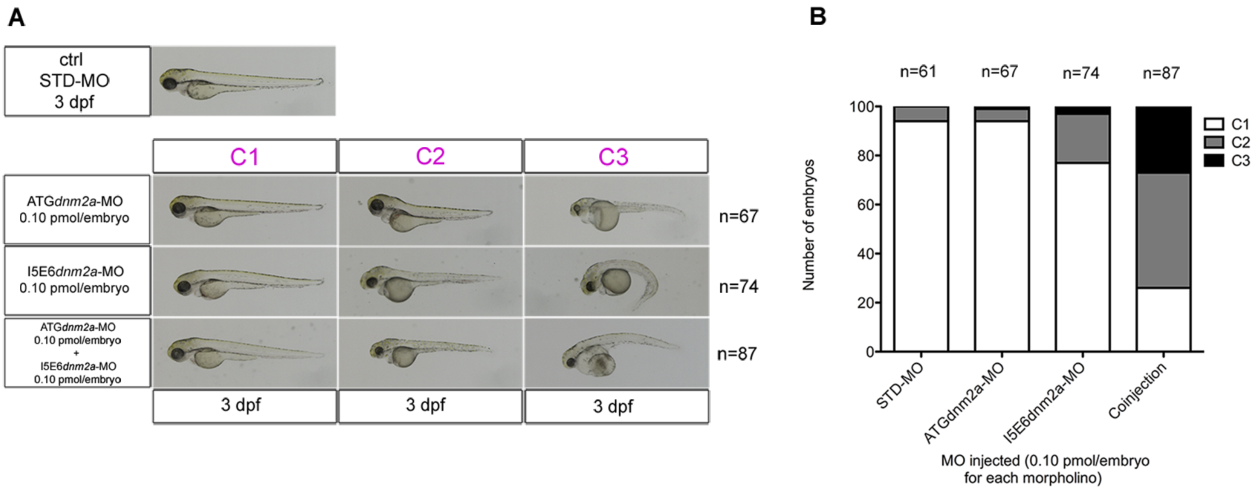

Supplementary Fig. 6. (A) Low doses (0.10 pmol/embryo) of ATG*dnm2a*-MO and I5E6*dnm2a*-MO were co-injected in the same embryos, compared with the injection of 0.10 pmol/embryo of STD-MO. (B) When combined, the morpholinos cause severe morphological alterations even at doses that were negligible on their own, confirming their targeting specificity.

A

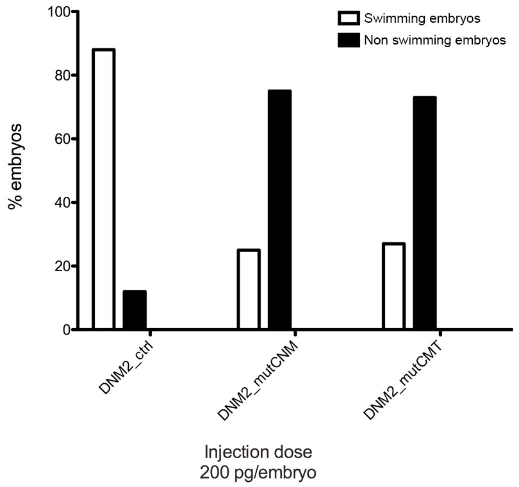

B

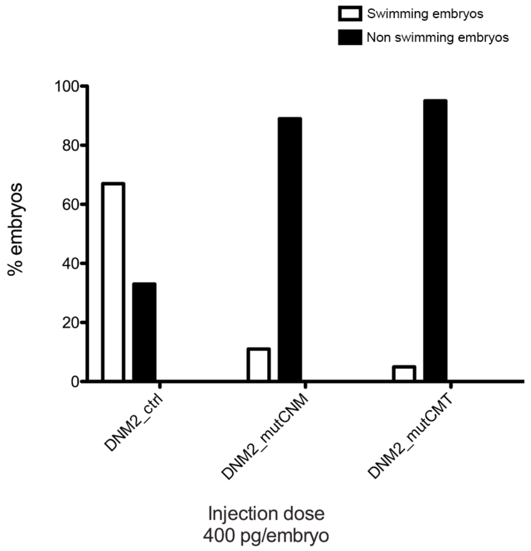

Supplementary Fig. 7. Graphs show percentage of embryos that present movement defects, or not, with injection of 200 pg and 400 pg of mRNA. 200 pg is the chosen concentration, in order to have a greater quantity of embryos that presented less movement defects.

Supplementary videos. Videos 1-3 show defective touch evoked responses in embryos injected with ATG*dnm2a*-MO (video 2), I5E6*dnm2a*-MO (video 3) in comparison to normal response in STD-MO (video 1) at 3 dpf. Videos 4-6 show defective touch evoked response in embryos injected with DNM2_mutCNM (video 5) and DNM2_mutCMT (video 6) in comparison to wild-type DNM2 (video 4) injected embryos, at 48 hpf.
